# De novo variants in *GABRA4* are associated with a neurological phenotype including developmental delay, behavioral abnormalities and epilepsy

**DOI:** 10.1038/s41431-024-01600-3

**Published:** 2024-04-02

**Authors:** Samin A. Sajan, Ralph Gradisch, Florian D. Vogel, Alison J. Coffey, Daria Salyakina, Diana Soler, Parul Jayakar, Anuj Jayakar, Simona E. Bianconi, Annina H. Cooper, Shuxi Liu, Nancy William, Ira Benkel-Herrenbrück, Robert Maiwald, Corina Heller, Saskia Biskup, Steffen Leiz, Dominik S. Westphal, Matias Wagner, Amy Clarke, Thomas Stockner, Margot Ernst, Akanchha Kesari, Martin Krenn

**Affiliations:** 1 Department of Pediatrics, University of Wisconsin School of Medicine and Public Health, Madison, WI USA; 2 Center for Physiology and Pharmacology, Medical University of Vienna, Vienna, Austria; 3 Department of Pathobiology of the Nervous System, Center for Brain Research, Medical University of Vienna, Vienna, Austria; 4 lllumina Clinical Services Laboratory, Illumina Inc., San Diego, CA USA; 5 Personalized Medicine and Health Outcomes Research, Nicklaus Children’s Hospital, Miami, FL USA; 6 Division of Genetics and Metabolism, Nicklaus Children’s Hospital, Miami, FL USA; 7 Department of Neurology, Division of Epilepsy, Nicklaus Children’s Hospital, Miami, FL USA; 8 Kaiser Permanente, San Diego, CA USA; 9 GeneDx, Gaithersburg, MD USA; 10 Mayo Clinic, Rochester, MN USA; 11 Kinderneurologisches Zentrum, Sana Kliniken Düsseldorf, Düsseldorf, Germany; 12 Medizinisches Versorgungszentrum für Gerinnungsdiagnostik und Medizinische Genetik Köln, Köln, Germany; 13 Zentrum für Humangenetik, Tübingen, Germany; 14 Center for Genomics and Transcriptomics (CeGaT), Tübingen, Germany; 15 Division of Neuropediatrics, Klinikum Dritter Orden, Munich, Germany; 16 Institute of Human Genetics, School of Medicine, Klinikum rechts der Isar, Technical University of Munich, Munich, Germany; 17 Department of Internal Medicine I, School of Medicine & Klinikum rechts der Isar, Technical University of Munich, Munich, Germany; 18 Department of Neurology, Medical University of Vienna, Vienna, Austria; 19 Comprehensive Center for Clinical Neurosciences & Mental Health, Medical University of Vienna, Vienna, Austria; 20 Present Address: Institute of Pharmacology and Toxicology, University of Zurich, Zurich, Switzerland

**Keywords:** Epilepsy, Neurodevelopmental disorders, Disease genetics, Next-generation sequencing

## Abstract

Nine out of 19 genes encoding GABA_A_ receptor subunits have been linked to monogenic syndromes characterized by seizures and developmental disorders. Previously, we reported the de novo variant p.(Thr300Ile) in *GABRA4* in a patient with epilepsy and neurodevelopmental abnormalities. However, no new cases have been reported since then. Through an international collaboration, we collected molecular and phenotype data of individuals carrying de novo variants in *GABRA4*. Patients and their parents were investigated either by exome or genome sequencing, followed by targeted Sanger sequencing in some cases. All variants within the transmembrane domain, including the previously reported p.(Thr300Ile) variant, were characterized in silico and analyzed by molecular dynamics (MD) simulation studies. We identified three novel de novo missense variants in *GABRA4* (NM_000809.4): c.797 C > T, p.(Pro266Leu), c.899 C > A, p.(Thr300Asn), and c.634 G > A, p.(Val212Ile). The p.(Thr300Asn) variant impacts the same codon as the previously reported variant p.(Thr300Ile) and likely arose post-zygotically as evidenced by sequencing oral mucosal cells. Overlapping phenotypes among affected individuals included developmental delay (4/4), epileptiform EEG abnormalities (3/4), attention deficits (3/4), seizures (2/4), autistic features (2/4) and structural brain abnormalities (2/4). MD simulations of the three variants within the transmembrane domain of the receptor indicate that sub-microsecond scale dynamics differ between wild-type and mutated subunits. Taken together, our findings further corroborate an association between *GABRA4* and a neurological phenotype including variable neurodevelopmental, behavioral and epileptic abnormalities.

## Introduction

Gamma-aminobutyric acid sub-type A receptors (GABA_A_Rs) are structurally heterogeneous ligand-gated anion channels contributing to inhibitory neurotransmission. These receptors are pentamers composed of different subunits encoded by 19 genes, thereby enabling the formation of a substantial diversity of receptor subtypes. The best-known assemblies are composed of two alpha (α), two beta (β), and one gamma (γ) subunit [1]. Disruption of GABA_A_R-mediated neurotransmission often results in epilepsy and neurodevelopmental phenotypes [2].

In mammalian brains, α4-containing GABA_A_Rs are expressed in neurons as well as glial cells and contribute to a wide diversity of pentameric assemblies, including some containing a delta (δ) subunit [3]. In both rodents and humans, expression has been shown to be highest in the dentate gyrus and the thalamus, followed by intermediate expression in the striatum, and lower expression in most other brain regions [4, 5]. α4-subunit containing receptors are mainly found in extrasynaptic regions and carry out inhibitory neurotransmission by tonic inhibition [6]. The exact composition of these receptors appears to be diverse [3], and structural studies indicate complex assembly rules and unusual arrangements [7]. While *Gabra4* knockout mice do not exhibit overt seizures, they are more susceptible to pentylenetetrazole induced seizures, and transcription profiling has shown alterations in the expression of genes involved in epilepsy, autism, and memory [8]. Moreover, a markedly increased expression of *GABRA4* was found following seizures, supporting its involvement in epileptogenesis [9].

Enrichment of both rare and common GABA_A_R variants increases the risk of developing common forms of epilepsy in a complex manner [10, 11]. GABAergic dysregulation due to polygenic factors is also implicated in the pathogenesis of neuropsychiatric conditions [12, 13]. Variants in a subset of GABA_A_R-encoding genes (*GABRA1-3*, *GABRA5*, *GABRB1-3*, *GABRG2*, *GABRD*) are associated with Mendelian disorders that comprise neurodevelopmental issues and epilepsy [14]. In addition to these genes, *GABRA4*, encoding the α4-subunit of the GABA_A_R, has recently been posited as a candidate gene based on our previous work in which we identified the p.(Thr300Ile) de novo missense variant in a patient with early-onset intractable epilepsy and developmental delay [15]. We further demonstrated that this variant behaves abnormally, which was also confirmed independently by another group [16]. Since then, no other patients with de novo variants in *GABRA4* and similar phenotypes have been reported.

Here, we provide additional evidence supporting the notion that *GABRA4* is a monogenic disease gene by reporting de novo missense variants in three additional patients with diverse neurodevelopmental, behavioral and epileptic phenotype features. Data from molecular dynamics (MD) simulations of all variants within the transmembrane domain (TMD), including the previously reported variant, indicated that they are likely to be pathogenic.

## Methods

### Genetic testing

#### Patient 1

Genetic analyses were performed as previously reported [15]. In brief, trio exome sequencing was performed using SureSelect Human All Exon Kit 60 Mb, V6 (Agilent, Santa Clara, California, USA) for exome enrichment, and libraries were sequenced on an Illumina HiSeq4000 system (Illumina, San Diego, CA, USA). The reported variant was confirmed by Sanger sequencing from blood and oral mucosa.

#### Patient 2

Sequencing libraries of the patient and the parents were prepared using either the Twist enrichment workflow (Twist Bioscience, San Francisco, CA) and a custom-design enrichment probe set (CeGaT ExomeXtra 1.0), or using the SureSelectXT workflow (Agilent, Santa Clara, CA) and the Human All Exon enrichment kit (version 6). Paired-end sequencing was performed on a NovaSeq instrument (Illumina, San Diego, CA). Trimmed raw reads were aligned to the human genome (hg19) with the Burrows-Wheeler Aligner. Average coverage on targets for trio analysis were 185x, 162x and 157x, respectively. Sequence variants were called (VarScan 2.4.2, CeGaT extended version or CeGaT stratacall) with a minimum variant allele frequency of 5%. Resulting variants were annotated with population frequencies from dbSNP, gnomAD (2.1/3.1) or ExAC (0.3.1), respectively, and an internal database (CeGaT), with functional predictions from dbNSFP, with publications from the Human Gene Mutation Database (HGMD) available at the time, and with transcript information from Ensembl, RefSeq, Gencode, and CCDS. Variants were filtered to remove frequent variants (MAF < 1.5%).

#### Patient 3

Sequencing was done via patient-parents trio clinical genome sequencing (cGS) using whole blood. This test was provided through the Illumina iHope program by the Illumina Clinical Services Laboratory as previously described [17]. CNVs greater than 10 kb were assessed using the read-depth based variant caller Canvas [18], whereas those less than 10 kb were determined using the split-read caller Manta [19]. Confirmation of the mosaic *GABRA4* missense variant was performed by targeted Sanger sequencing using DNA from a newly drawn sample of whole blood and oral mucosal cells obtained from a buccal swab only from the patient. Parental samples were not sequenced by Sanger.

#### Patient 4

Clinical exome sequencing of the patient and the mother was carried out. Briefly, the exonic regions and flanking splice junctions of the genome were captured using the IDT xGen Exome Research Panel v1.0 (Integrated DNA Technologies, Coralville, IA). Massively parallel (NextGen) sequencing was done on an Illumina system with 100 bp or greater paired-end reads. Reads were aligned to the human genome build GRCh37/UCSC hg19, and analyzed for sequence variants using a custom-developed analysis tool. Targeted Sanger sequencing was used to confirm the absence of the *GABRA4* variant in the father. Additional sequencing technology and variant interpretation protocol have been previously described [20].

### In silico molecular characterization

The ɑ4β3δ-containing receptor in a pre-open state (PDB ID: 7QN9) [7] was inserted in a complex equilibrated lipid membrane [21], and used as the basis for molecular dynamics (MD) simulations. Three independent replicates were simulated per condition (ɑ4Wild-type (WT), ɑ4Pro266Leu, ɑ4Thr300Asn, ɑ4Thr300Ile) using Gromacs 2022.3 [22] to investigate the molecular and dynamic behavior of mutated ɑ4-containing GABA_A_Rs relative to the WT on a time scale of 0.5 microseconds (μs). Additional methods are provided as supplement.

## Results

### Clinical and genetic findings

#### Patient 1 (previously reported) [15]

A detailed clinical assessment of this seminal patient with a de novo variant in *GABRA4* has been reported previously [15]. In summary, this is currently an 8-year-old white female from Germany born at term after an uneventful pregnancy. Developmental (predominantly speech) delay was first evident at 2 years of age. At 3.5 years of age she started experiencing seizures that occurred in clusters during sleep. At this time, she weighed 14.1 kg (25th percentile), had a length of 95 cm (11th percentile), and a head circumference of 49 cm (25th percentile). Seizures were clinically characterized by sudden awakening, subsequent unresponsiveness, and shaking/ballistic movements of the left lower extremity. EEG recordings indicated a seizure of right frontal origin (Supplementary Fig. 1). Based on the comprehensive diagnostic work-up, non-lesional focal epilepsy was diagnosed. Due to seizure exacerbation she required multiple anti-seizure medication trials including temporary treatment in an intensive care unit. Following initial seizure clusters, the patient remained seizure-free for 2 years while on brivaracetam and lamotrigine. Recent EEG recordings showed no more epileptiform discharges but revealed high-amplitude generalized, rhythmic theta activity with a bifrontal maximum. The patient still exhibits marked speech delay, verbal dyspraxia and attention deficit but no developmental plateauing or regression. Additionally, she has insomnia that is not responsive to melatonin treatment.

Trio exome sequencing revealed the de novo missense variant c.899 C > T, p.(Thr300Ile) in *GABRA4* (NM_000809.4) in the second transmembrane domain (TMD). This variant is absent in gnomAD and is predicted as pathogenic by both REVEL (score of 0.95) and AlphaMissense (score of 0.9945). Previous electrophysiological characterization of this variant showed that it caused accelerated desensitization and lack of endogenous seizure-protective neurosteroid function. This variant was present in a mosaic state in whole blood at a frequency of 17% (26 variant reads out of 155 reads), an observation that was confirmed in buccal epithelial cells by targeted Sanger sequencing [15].

#### Patient 2

This patient is a 9-year-old White female from Germany with an unremarkable family history. After an uncomplicated pregnancy, she was born spontaneously at 40 weeks of gestation with a weight of 3590 g (61st percentile), length of 51 cm (37th percentile), and a head circumference of 34 cm (25th percentile). APGAR score was 9/10/10. The early postnatal period was unremarkable. She started to walk unaided at 19 months and used word combinations at 33 months. Medical attention was first sought at around three years of age due to global developmental delay. However, there was no plateauing or regression. A neuropsychological assessment at 3 years of age indicated intellectual disability (IQ < 55) with marked deficits in all tested domains (motor development, hand dexterity, perception, speech, language comprehension, social skills). She was only capable of using 10 single words and forming her first two-word sentences.

Behavioral abnormalities included impaired social interaction, aggressive behavior, attention deficits, repetitive behavior/language, and a tendency towards rituals. Speech development was markedly delayed, and the patient’s eye contact was non-modulating in social situations consistent with autism spectrum disorder (according to DSM-5 criteria). She also experienced disrupted sleep, waking up several times per night with intermittent episodes of crying. The patient has received speech and occupational therapy on a regular basis.

Physical exam at 3.5 years showed a broad face, upwardly slanting outer corners of the eyes, hypotelorism, narrow eyelid openings, a prominent forehead, a full lower lip, an inverted left nipple and a single café-au-lait spot measuring 1 cm over the right scapula. Her BMI was at the 97th percentile for age and sex, indicating obesity. A neurological exam revealed unsteady, broad-based gait, but no focal deficits. Although the patient has not experienced apparent epileptic seizures so far, EEG recordings showed focal epileptiform discharges with sharp waves with a right fronto-centro-temporal maximum (Supplementary Fig. 2). Anti-seizure medication including sultiame and clobazam did not show any benefit. Brain MRI at three years old revealed subtle subcortical signal hyperintensities which were maximal in the parietal and occipital lobes, potentially suggestive of delayed myelination (Supplementary Fig. 3).

Clinical trio exome sequencing identified the heterozygous de novo variant c.797 C > T, p.(Pro266Leu) in *GABRA4* (Supplementary Fig. 4) with a variant allele frequency of 63% (36 variant reads out of 57 reads), indicating that this was a heterozygous variant. This variant is in the first TMD, absent from gnomAD, and predicted as pathogenic by both REVEL (score of 0.94) and AlphaMissense (score of 0.9963).

#### Patient 3

This patient is currently a 4-year-old white male of Mexican/Irish/French ancestry on the paternal side and English/German on the maternal side. After an uncomplicated pregnancy, he was born at 40 weeks of gestation via C section with a weight of 3742 g (64th percentile), length of 53.3 cm (88th percentile), and a head circumference of 37 cm (75th percentile). Early motor development was unremarkable, but shortly thereafter, hypotonia and increasing delays were noted. Specifically, he was able to grab toys at 4 months, lie prone with head up at 5.5 months, sit independently at 7.75 months, and put food in his mouth by 11.5 months. Between 6 and 20 months he gradually lost his milestones and is currently unable to roll, sit, or stand, and has poor head control. He does not understand commands and is unable to eat, having failed multiple follow-up studies.

Atonic and myoclonic seizures began at six to seven months of age and he was treated with multiple anti-seizure medications without satisfactory improvement. Ketogenic diet worsened both seizures and development, and was therefore discontinued at 15 months of age. Initial EEGs showed disorganized background with mild slowing, and different seizure types of multifocal origin. Subsequent EEGs revealed slow spike-and-wave discharges and multifocal spikes (Supplementary Fig. 5). Brain MRI at 18 months showed an increased FLAIR signal in the right frontal lobe white matter (Supplementary Fig. 6) which was initially interpreted as a focal cortical dysplasia (FCD), and a linear area of enhancement in the left frontal lobe, which was most likely related to a developmental venous anomaly. Increased T2-weighted signal was also seen in the pons, most likely thought to be focal hypomyelination.

At 2.5 years he had brain surgery to remove the right frontal focal brain lesion. However, pathology results were consistent with a mild malformation of cortical development with oligodendroglial hyperplasia in frontal lobe epilepsy (MOGHE), an entity characterized by subcortical oligodendroglial hyperplasia in young patients with frontal lobe epilepsy [23]. Due to ongoing treatment resistance, the patient subsequently underwent a corpus callosotomy which eventually reduced the seizure frequency. Based on seizure semiology and EEG findings, multifocal epilepsy was diagnosed. Hence, the MOGHE lesion was no longer considered the primary cause of the seizure disorder. The patient is currently globally delayed, not aware of his surroundings, non-verbal, and has axial hypotonia and choreiform movements.

Initially, a chromosomal microarray revealed a duplication of 862 kilobases (kb) on chromosome 2q13 (hg19: chr2:110504318-111365996) impacting eighteen genes of which 7 are protein-coding. None of the eighteen genes is currently associated with human disease in the OMIM database except for *NPHP1* which causes autosomal recessive nephronophthisis with or without Joubert syndrome that are not consistent with this patient’s features. Subsequent trio clinical genome sequencing showed that this duplication was inherited from the unaffected mother and was therefore not considered to be causative. Clinical genome sequencing identified the de novo c.899 C > A, p.(Thr300Asn) variant in the second TMD of *GABRA4* at a variant allele frequency of 16% (7 variant reads out of 44 reads), indicating somatic mosaicism (Supplementary Fig. 7). This was confirmed by targeted Sanger sequencing using a new blood sample and oral mucosa cells (Supplementary Fig. 8). Quantification of the Sanger peak heights showed that this variant was present at approximately 9.2% in blood and 19.8% in oral mucosa, respectively (Supplementary Table 1). This variant is absent from gnomAD and predicted to be pathogenic by both REVEL (score of 0.85) and AlphaMissense (score of 0.9921).

#### Patient 4

This patient is currently 10-year-old white male who was born via C-section after 38.4 weeks of gestation with an APGAR score of 8/9. He was small for gestational age with a birth weight of 1940 g (1st percentile) due to intrauterine growth restriction (IUGR). He was admitted to the NICU due to hypoglycemia as well as reflux and problems establishing oral feeds. Developmental milestones were markedly delayed, and he was diagnosed with borderline intellectual disability including learning and speech delays. He first rolled at 5.5 months, sat at six months, crawled and walked with support at 12 months, started walking at 20 months and achieved independent walking at 24 months. No focal abnormalities were noted, although there was reduced muscle tone. Brain MRI at two years of age was normal. At 11 years, he weighed 31.9 kg (27th percentile) and measured 142.2 cm in height (45th percentile). The head circumference at 9 years and 7 months was 50.7 cm (6th percentile).

Additional features included ptosis, Marcus Gunn jaw winking syndrome, tics, microcephaly, downslanting palpebral fissures (also in the patient’s mother), strabismus, prominent low-set ears with pronounced bilateral Darwin’s tubercles, smooth philtrum, puffy proximal phalanges, prominent dimples on all metatarsals with tapered appearance, a single transverse crease on the right, prominent fetal pads, bilateral 2,3 toe syndactyly (also seen in the mother), medially deviated great toes, eczema, and dimples on both elbows. The patient also had disruptive mood behavior, attention deficit hyperactivity disorder, and autism spectrum disorder. Subsequently, his behavioral abnormalities required admission to a residential facility for violent and aggressive behavior. He has not had any seizures and the awake and asleep EEG was normal (Supplementary Fig. 9). At last follow-up at 10 years old, ID was still in the mild range.

Clinical exome sequencing of the patient and the mother identified the heterozygous variant c.634 G > A, p.(Val212Ile) in *GABRA4* in the extracellular domain (ECD) only in the patient (Supplementary Fig. 10). The variant allele frequency of the variant was 46% (32 variant reads out of 69 reads) indicating that it was heterozygous. This variant was not detected in the father by targeted Sanger sequencing (Supplementary Fig. 11), suggesting a de novo occurrence. The variant is absent from gnomAD and has a REVEL score of 0.43 which is inconclusive about its predicted pathogenicity, and an AlphaMissense score of 0.223 which is considered benign. This patient also had the maternally inherited duplication on chrXq25-q26.1 of 668 kb (hg19: chrX:128673252-129338967) affecting twelve genes of which eleven are protein-coding. Of those, seven are associated with at least one disorder in the OMIM database. However, the features of this patient are not consistent with any of these disorders. Note that some features of this proband do overlap with those of the autosomal dominant disorder caused by pathogenic variants in the *ZDHHC9* gene, such as developmental delay, downslanting palpebral fissures, and behavior issues. However, the mechanism of disease through which this gene causes disease is haploinsufficiency/loss-of-function whereas this patient’s CNV duplicates this entire gene and is therefore not expected to result in loss-of-function. Furthermore, no similar duplications have been reported in other patients with features resembling those of this patient. For these reasons, this CNV is unlikely to be contributing to this patient’s phenotype.

The major features of all four patients are summarized in Table 1.

**Table 1:** Summary of main genetic and clinical findings of the four patients with de novo missense variants in *GABRA4*.

	Patient 1 [15]	Patient 2 (this study)	Patient 3 (this study)	Patient 4 (this study)
Gender	Female	Female	Male	Male
Current age	8 years	9 years	4 years	10 years
*GABRA4* variant (NM_000809.4)	c.899 C > T, p.(Thr300Ile)	c.797 C > T, p.(Pro266Leu)	c.899 C > A, p.(Thr300Asn)	c.634 G > A, p.(Val212Ile)
Confirmed de novo	Yes	Yes	Yes	No
Heterozygous/mosaic	Mosaic	Heterozygous	Mosaic	Heterozygous
In silico predictions	REVEL: pathogenic AlphaMissense: pathogenic	REVEL: pathogenic AlphaMissense: pathogenic	REVEL: pathogenic AlphaMissense: pathogenic	REVEL: uncertain AlphaMissense: benign
Epileptic seizures	Yes	No	Yes	No
Seizure types	Sleep-related hypermotor seizures, BTCS	N/A	Atonic, myoclonic and focal seizures, BTCS	N/A
EEG	Ictal and interictal epileptiform discharges in right frontal lobe	Sharp waves with right fronto-centro-temporal maximum	Disorganized background, slow spike-and-wave discharges, multifocal spikes	Normal (awake and asleep)
Anti-seizure treatment	LCM, LEV, PB, ZNS, VPA, BRV, LTG	STM, CLB	Multiple ASM, ketogenic diet, epilepsy surgery, callosotomy	N/A
Structural brain abnormalities	Normal MRI	MRI: White matter lesions (parietal, occipital and frontal lobe) indicating delayed myelination	MRI: suspected FCD, delayed myelination; Histology: MOGHE	Normal MRI
Developmental delay	Yes, predominantly speech	Yes, global	Yes, global	Yes, global
Intellectual disability	Borderline	Yes, IQ < 55	Yes, severe (non-verbal)	Borderline
Additional neurological/ behavioral abnormalities	Dyspraxia, deficits affecting attention, executive function, language comprehension, insomnia	ASD with impaired social interaction, aggressive behavior, attention deficits, repetitive behavior/language	Truncal and axial hypotonia, choreiform movements	ADHD and ASD with learning disability, tics, disruptive mood behavior

*ADHD* attention deficit hyperactivity disorder, *ASD* autism spectrum disorder, *ASM* anti-seizure medication, *BRV* brivaracetam, *BTCS* bilateral tonic-clonic seizures, *CLB* clobazam, *EEG* electroencephalography, *FCD* focal cortical dysplasia, *IQ* intelligence quotient, *LCM* lacosamide, *LEV* levetiracetam, *LTG* lamotrigine, *MOGHE* mild malformation of cortical development with oligodendroglial hyperplasia in frontal lobe epilepsy, *MRI* magnetic resonance imaging, *N/A* not applicable, *PB* phenobarbital, *STM* sultiame, *VPA* valproate, *ZNS* zonisamide.

### Structural analysis and MD simulations

**Fig. 1:**
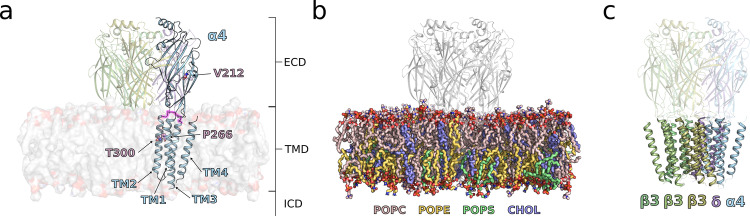
Structural basis for the MD simulations of α4 variants. **A** Side view of GABRα4β3δ embedded in a complex lipid bilayer. The α4 subunit, as derived from GABRα4β3δ cryo-EM structure (PDB ID: 7QN9) [7], is colored in skyblue and highlights the ECD and TMD. The three β3 and one δ subunits, protruding through the membrane, are colored in three shades of green and mauve, respectively. The linker segment connecting TM2 and TM3 is highlighted in faded pink. Amino acids that vary in the four patients are depicted in sticks and colored in mauve. **B** Lipid composition in sticks used for the MD simulations. POPC:1-palmitoyl-2-oleoyl-sn-glycero-3-phosphocholine; POPE: 1-palmitoyl-2-oleoyl-sn-glycero-3-phosphoethanolamine; POPS: 1-palmitoyl-2-oleoyl-sn-glycero-3-phospho-L-serine, CHOL: cholest-5-en-3β-ol, colored in rose, yellow, green, and blue, respectively. **C** Overview of the TMD arrangement. While the ECD is transparent, the TMD is kept opaque. The α4 subunit is sandwiched between the δ and a β3 subunit. Subunits are colored as in (**A**).

The variants detected in patients 1 p.(Thr300Ile), 2 p.(Pro266Leu) and 3 p.(Thr300Asn) are in the TMD, whereas the variant in patient 4 p.(Val212Ile) is in the ECD (Fig. 1A). We and others have previously reported that the human α4-subunit of GABA_A_R displays different electrophysiological properties in recombinantly expressed receptors compared to the rodent versions, and the human α4-subunit is exceedingly difficult to express in-vitro [15, 24].

To characterize the defects in GABA_A_R function of the TMD variants of the patients, we used MD simulations to investigate receptor motion with high temporal and spatial resolution. This allowed for the detection of molecular features resulting from the variants and their consequences for GABA_A_R dynamics at the single receptor level. We utilized the α4β3δ GABA_A_R cryo-EM structure which was co-resolved with histamine and in a pre-open state (PDB ID: 7QN9) [7] and we embedded the receptor into a near physiological complex membrane (Fig. 1A-C). The ECD variant p.(Val212Ile) is not suitable for sub-microsecond timescale investigation and was therefore not investigated.

**Fig. 2:**
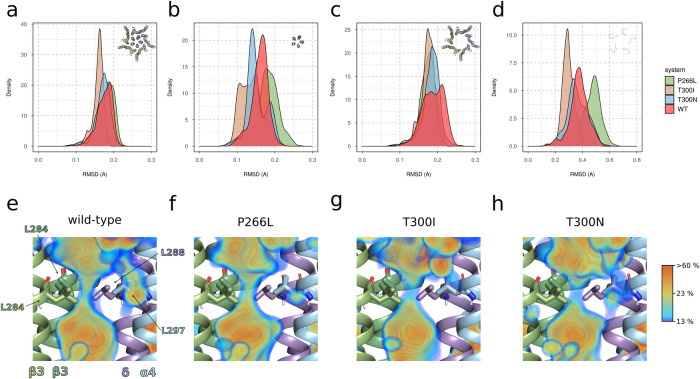
Results of the MD simulations, pooled for each variant. The distribution of root mean square deviation (RMSD) in Ångstrom units (Å) are computed for (**A**) all transmembrane helices, (**B**) helix 2 alone, (**C**) helices 1, 3, and 4, and (**D**) for the linker segment between helices 2 and 3 (coupling region). The individual variants are color-coded as defined in the color legend: wild type (WT): red; P266L: green; T300I: salmon; T300N: light blue. Average spatial occupancy of water at the level of the activation gate which is formed by a hydrophobic leucine ring α4: L297, β3: L284; δ: L288 computed for (**E**) WT, (**F**) P266L, (**G**) T300I, and (**H**) T300N. One β3 TMD is hidden to facilitate a front view of the gate. TMD and side chains are shown in cartoon and stick representation, respectively. Color coding is according to Fig. 1A, C.

MD simulations revealed distinct signatures of transmembrane domain behavior for each of the three TMD variants compared to wild type (Fig. 2A−C). The p.(Thr300Ile) and p.(Thr300Asn) variants, both affecting the same amino acid in the second transmembrane alpha-helix (TM2), showed an overall less dynamic TMD as suggested by a smaller root mean square deviation (RMSD) of the whole domain (Fig. 2A) and especially the second and pore forming alpha helix TM2 (Fig. 2B). In the case of p.(Thr300Ile), and to a lesser extent for p.(Thr300Asn), the linker between TM2 and TM3 was less mobile (Fig. 2D). Thus, these variants resulted in a generally more rigid (“stiffer”) TMD. Additionally, the average water occupancy next to the channel activation gate was reduced for these two variants (Fig. 2G, H) compared to wild type (Fig. 2E). These findings support the electrophysiological properties of the p.(Thr300Ile) variant which we reported previously namely higher desensitization due to faster channel inactivation as discussed further below [15]. In contrast, the p.(Pro266Leu) variant, located in the first alpha-helix (TM1), featured a more dynamic behavior compared to wild type because of the higher mobility of the pore forming TM2 α-helices but not of the other TM helices as evidenced by a shift to a larger RMSD (Fig. 2B). A similar change was observed for the coupling helices (Fig. 2D). However, none of the other TM helices exhibited an elevated dynamic behavior with respect to wild type. In keeping with the increased dynamics of TMD caused by p.(Pro266Leu), a slightly increased average water occupancy next to the activation gate was observed (Fig. 2F). Thus, MD simulations of α4β3δ receptors unravel unique features for each *GABRA4* TMD variant in comparison to wild type.

## Discussion

Genetic variants in GABA_A_R-encoding genes have long been recognized to be associated with epilepsies and neurodevelopmental disorders, as either monogenic/Mendelian or as polygenic risk factors [14]. Previously, we reported one patient with epilepsy with a de novo missense variant in *GABRA4*, thus making this a candidate gene for monogenic epilepsy [15]. In the present study, we describe three additional unrelated patients with de novo missense variants in *GABRA4* displaying diverse neurodevelopmental and behavioral abnormalities that may or may not be accompanied by seizures. The clinical picture observed in the four patients is broad but overlaps with the previously reported patient as well as with patients who harbor pathogenic variants in other GABA_A_R subunit genes. The core phenotype includes developmental delay, intellectual disability, behavioral abnormalities and epilepsy.

While all the reported patients had a neurodevelopmental disorder, three had epileptiform EEG changes or overt epileptic seizures. Patients 1, 2, and 3, all of who had epileptic features, had variants in the TMD whereas patient 4, who did not experience seizures and had a normal EEG, harbored a variant within the ECD. Consistent with this observation, the TMD of other GABA_A_R subunit genes also constitutes a hot spot for epilepsy-related variation [25].

The only reported individual without any epileptic features (patient 4) displayed a complex phenotype, and it is unclear which traits can be attributed to the *GABRA4* variant. Some of his physical findings such as the 2,3 toes syndactyly may be familial and not associated with his neurodevelopmental findings. And since this patient’s father was tested only by targeted Sanger and not by exome sequencing, non-paternity cannot be completely ruled out. Moreover, in silico prediction of this variant by REVEL is inconclusive whereas by AlphaMissense is benign. Thus, the overall contribution of this variant to the phenotype observed in patient 4 remains uncertain.

Structural brain abnormalities were found in patients 2 and 3, including histologically confirmed MOGHE and signs of delayed myelination, whereas in patient 1, focal epilepsy was suspected only based on clinical and EEG findings. Although the underlying molecular mechanisms remain to be elucidated, it is known that germline variants in other GABA_A_R genes can cause monogenic focal/multifocal epilepsy [26]. With respect to MOGHE, which is an oligodendroglial pathology, it is noteworthy that α4-containing receptors are also expressed in several glial cell types [4]. However, the role of *GABRA4* expression in the prenatal brain and in neuroglial precursor cells remains unexplored at this time.

The *GABRA4* gene shows a statistically significant depletion of missense variation in the general population, particularly in the region of the gene that encodes amino acids 1-334 where all the de novo variants reported here are located [27]. Interestingly, the variant seen in patient 3, p.(Thr300Asn), affects the same codon as the previously published p.(Thr300Ile) variant in patient 1. Both variants are likely a result of early post-zygotic events because they were observed at an allele fraction of approximately 16−17% in whole blood, which was confirmed in ectodermal tissue (oral mucosa). Somatic mosaicism of pathogenic variants has been observed in many genes that cause monogenic epilepsy including in some GABA_A_R subunit genes, ranging from 11.7 to 18.6% in whole blood [28, 29]. T300 is located in the second and pore forming transmembrane helix within a conserved T-T-I/L motif where several de novo variants in other GABA_A_R subunits resulting in epilepsy have been reported at equivalent positions [25]. Furthermore, functional evaluation of the p.(Thr300Ile) variant through electrophysiological recordings in *Xenopus laevis* oocytes showed that this variant caused accelerated desensitization kinetics indicative of faster channel inactivation, and a lack of neurosteroid function [15]. Later, another group reported a partial gain-of-function effect based on higher peak current amplitudes and a left shift in GABA dose response suggesting a complex molecular change-of-function [16]. Due to the developmental switch of GABAergic signaling from chiefly excitatory to chiefly inhibitory around the time of birth, it cannot be predicted how molecular gain or loss of function properties precisely impact the prenatal and postnatal circuitries [30].

Molecular dynamics simulation revealed unique behavior signature for each TMD variant relative to wild type receptors. No MD simulations were performed on the ECD variant, as these would not be informative at the accessible time scale of the simulations and the assumption of an allosteric communication mechanism for the ECD to the pore forming TM2. The p.(Pro266Leu) variant, located in TM1, resulted in an elevated mobility of TM2 and an associated increase in the average water occupancy at the activation gate. Both phenomena could hint towards a gain-of-function effect (such as faster activation due to extended time in the open state) relative to the two variants affecting p.Thr300 in TM2. Both the p.(Thr300Ile) and p.(Thr300Asn) variants exhibited reduced mobility (slow systems), especially in the coupling helix regions. This may be indicative of longer time spent in the closed state and hence potentially loss of function which would be in line with our previous electrophysiological characterization of the p.(Thr300Ile) variant in *Xenopus* oocytes. Specifically, this variant resulted in higher desensitization indicative of faster channel inactivation as was demonstrated by markedly reduced responses evoked by GABA as well as by the endogenous neurosteroid tetrahydrodeoxycorticosterone (THDOC) compared to wild type channels [15].

MD simulation data must be interpreted with caution due to some inherent limitations. Finite simulation time (here sub-microsecond) combined with a relatively low number of replicates (*n* = 3) may not allow detecting effects that occur rarely or at longer time scales. Furthermore, since the first cryo-EM structure with multiple α4β3δ receptors and a variety of ligands was published recently [7], our simulations were performed on receptors stemming from heterologous expression systems rather than native GABA_A_Rs that might differ in subunit composition and arrangement. Qualitatively, our MD simulation data correlate well with experimental findings because the “stiffer” TMD with lower water occupancy observed for p.(Thr300Ile) and p.(Thr300Asn) is consistent with a stabilized closed-desensitized state, while the more dynamic TMD and higher water occupancy observed for p.(Pro266Leu) is consistent with experimental in vitro findings of orthologous variants. Specifically, variants that impact the equivalent proline residue in subunits α1, β2, β3 and γ2 are known to be associated with epilepsy and result in a change-of-function [31–36]. In contrast, the p.(Val212Ile) variant in the ECD is localized in a critical region of the domain which has been implicated in the coupling between ligand binding and downstream effects, and thus also likely to display a molecular change-of-function.

In summary, we provide genetic evidence complemented by MD simulation data to associate de novo variants in *GABRA4* with a spectrum of neurodevelopmental, behavioral and epileptic phenotypes. In the future, additional cases need to be collected to fully delineate the clinical spectrum related to *GABRA4*. Moreover, biochemical and electrophysiological characterizations of identified variants as well as studies that clarify *GABRA4* expression patterns in prenatal brain development will be crucial to better appreciate the underlying pathomechanisms.

### Supplementary information

Supplemental figures 1-11, supplementary table 1, and supplementary methods

## Data Availability

The patients and the *GABRA4* variants reported here have been submitted to the Leiden Open Variation Database (LOVD) which can be accessed at: https://lovd.nl/GABRA4. The computational source data of Fig. 2 as well as each start-end structure of GABRα4β3δ, extracted from all trajectories, are available at Zenodo (https://zenodo.org) with the: 10.5281/zenodo.10601195. The cryo-EM structure of the PDB entry (7QN9) of GABRα4β3δ in a pre-open/closed state with a resolution of 2.90 angstroms [https://www.rcsb.org/structure/7QN9] was used as a starting point to generate MD input structures. Additional data may be provided upon reasonable request from the corresponding author.
