# Supplementary material for: De novo variants in GABRA4 are associated with a neurological phenotype including developmental delay, behavioral abnormalities and epilepsy
Source: Eur J Hum Genet. 2024 Apr 2;32(8):912–9. doi: 10.1038/s41431-024-01600-3 (PMC11291759; doi:10.1038/s41431-024-01600-3)
Supplement: Supplementary file 1 — Supplemental figures 1-11, supplementary table 1, and supplementary methods [file 41431_2024_1600_MOESM1_ESM.pdf]

SUPPLEMENTARY MATERIAL

De novo variants in *GABRA4* cause variable neurodevelopmental, behavioral and epilepsy-related abnormalities

Samin A. Sajan[*1], Florian D. Vogel[*2], Alison J. Coffey[3], Daria Salyakina[4], Diana Soler[4], Parul Jayakar[5], Anuj Jayakar[6], Simona E. Bianconi[7], Annina H. Cooper[7], Shuxi Liu[8], Nancy William[9], Ira Benkel-Herrenbrück[10], Robert Maiwald[11], Corina Heller[12], Saskia Biskup[12,13], Steffen Leiz[14], Dominik S. Westphal[15], Matias Wagner[15], Ralph Gradisch[16], Amy Clarke[16], Thomas Stockner[16], Margot Ernst[2], Akanchha Kesari[3], Martin Krenn[17,18]

1 Department of Pediatrics, University of Wisconsin School of Medicine and Public Health, Madison, WI, USA

2 Department of Pathobiology of the Nervous System, Center for Brain Research, Medical University of Vienna, Vienna, Austria

3 Illumina Clinical Services Laboratory, Illumina Inc., San Diego, CA, USA

4 Personalized Medicine and Health Outcomes Research, Nicklaus Children's Hospital, Miami, FL, USA

5 Division of Genetics and Metabolism, Nicklaus Children's Hospital, Miami, FL, USA

6 Department of Neurology, Division of Epilepsy, Nicklaus Children's Hospital, Miami, FL, USA

7 Kaiser Permanente, San Diego, CA, USA

8 GeneDx, Gaithersburg, MD, USA

9 Mayo Clinic, Rochester, MN, USA

10 Kinderneurologisches Zentrum, Sana Kliniken Düsseldorf, Düsseldorf, Germany

11 Medizinisches Versorgungszentrum für Gerinnungsdiagnostik und Medizinische Genetik Köln, Köln, Germany

12 Zentrum für Humangenetik, Tübingen, Germany

[13] Center for Genomics and Transcriptomics (CeGaT), Tübingen, Germany

[14] Division of Neuropediatrics, Klinikum Dritter Orden, Munich, Germany

[15] Institute of Human Genetics, School of Medicine, Klinikum rechts der Isar, Technical University of Munich, Munich, Germany

[16] Center for Physiology and Pharmacology, Medical University of Vienna, Vienna, Austria

[17] Department of Neurology, Medical University of Vienna, Vienna, Austria

[18] Comprehensive Center for Clinical Neurosciences & Mental Health, Medical University of Vienna, Vienna, Austria

[*] Contributed equally

Correspondence to: Martin Krenn, MD, PhD; Department of Neurology, Medical University of Vienna, Vienna, Austria: martin.krenn@meduniwien.ac.at

Supplementary figure 1. EEG of patient 1 reveals (A) a background EEG with a high-amplitude, generalized rhythmic theta activity, and (B) a seizure originating from the frontal region, initiating with a rhythmic alpha activity, subsequently showing a rapid generalization (not shown), clinically accompanied by impaired consciousness and oral automatisms.

A

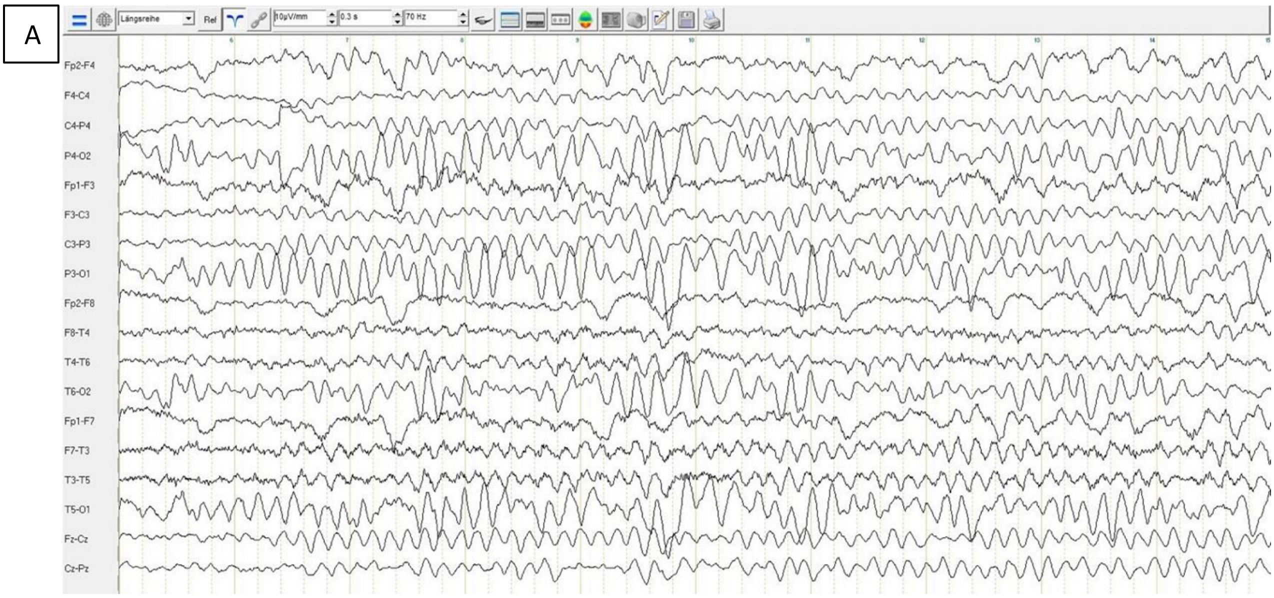

B

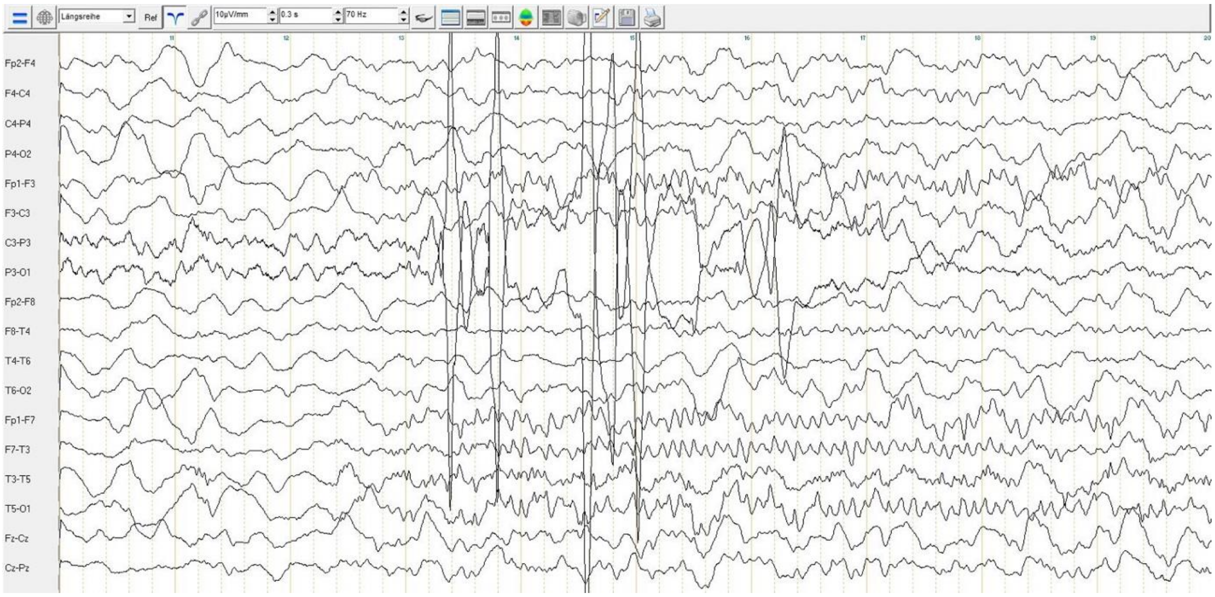

Supplementary figure 2. EEG of patient 2 demonstrates (A+B) singular and repetitive spikes and sharp slow waves with a right fronto-centro-temporal maximum, increasing in frequency and amplitude during sleep.

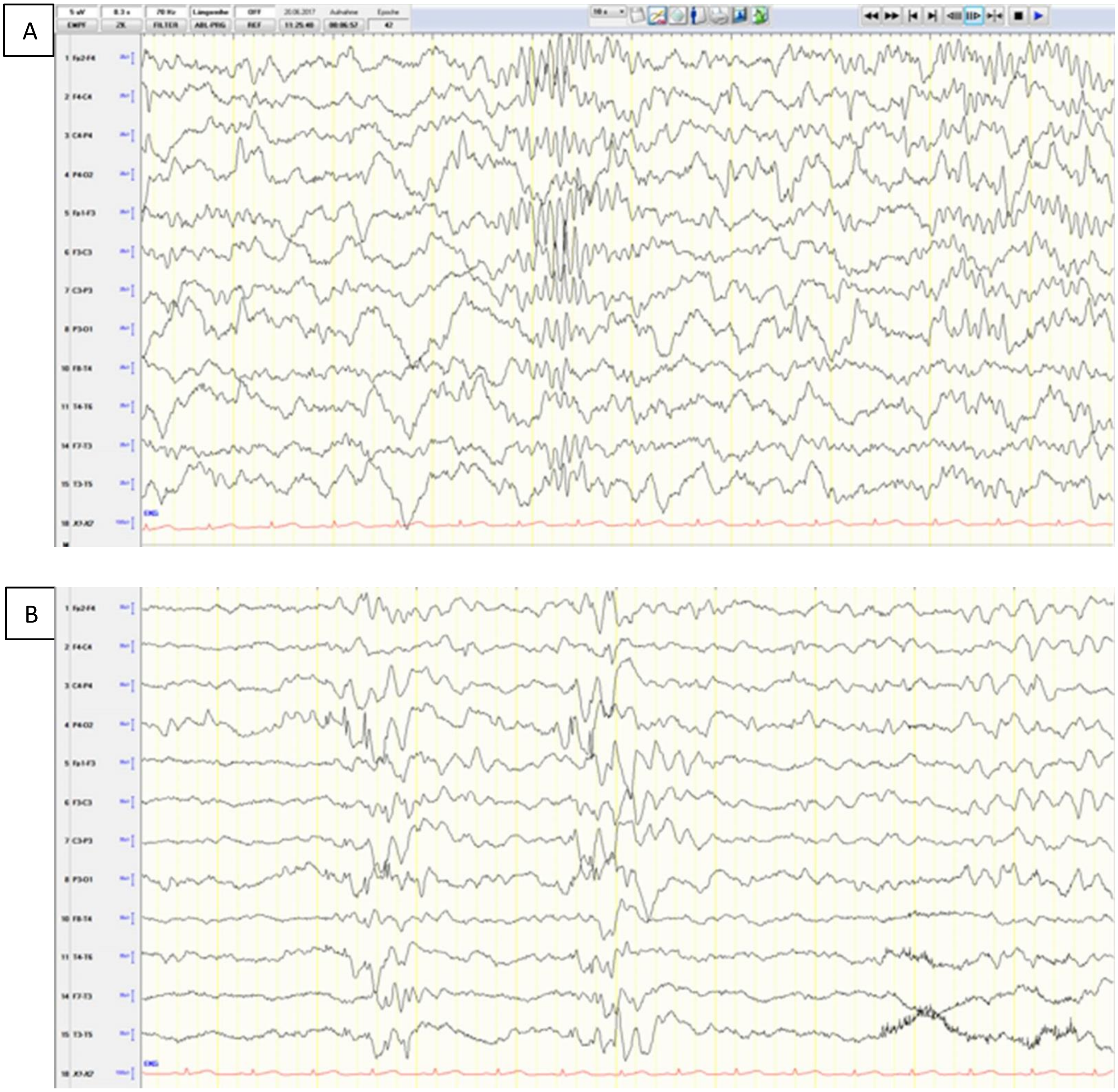

Supplementary figure 3. Coronal T2-weighted brain MRI of patient 2 showing subtle subcortical signal hyperintensities (indicated by white arrows) in parietal, occipital, temporal and frontal lobes (maximum parietal and right occipital lobes), potentially suggestive of delayed myelination.

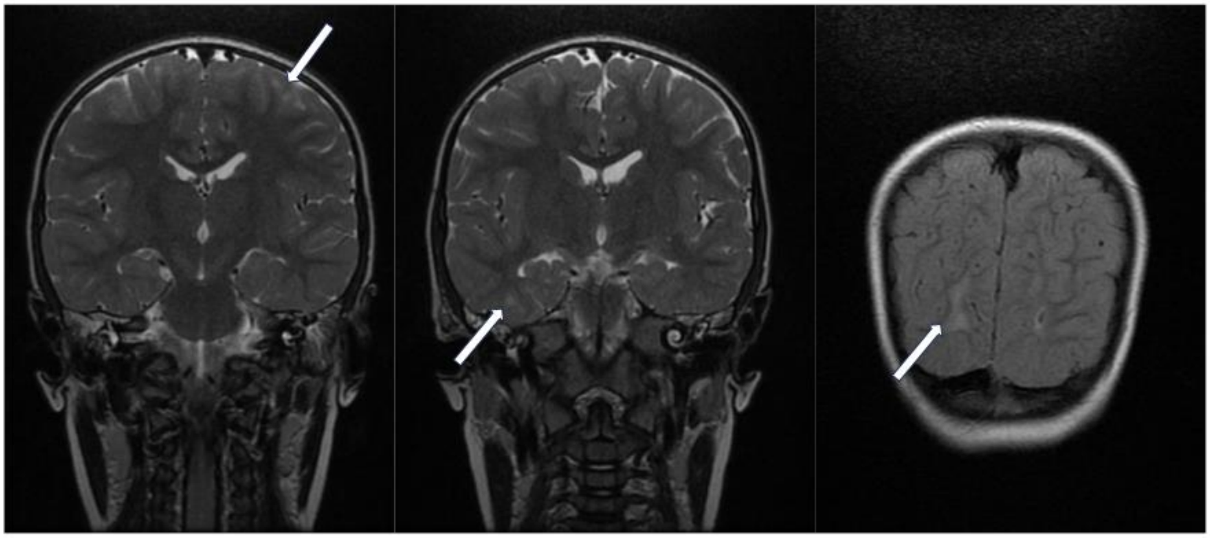

Supplementary figure 4. Integrated Genome Browser (IGV) views of the c.797C>T, (p.P266L) variant in patient 2 and the parents.

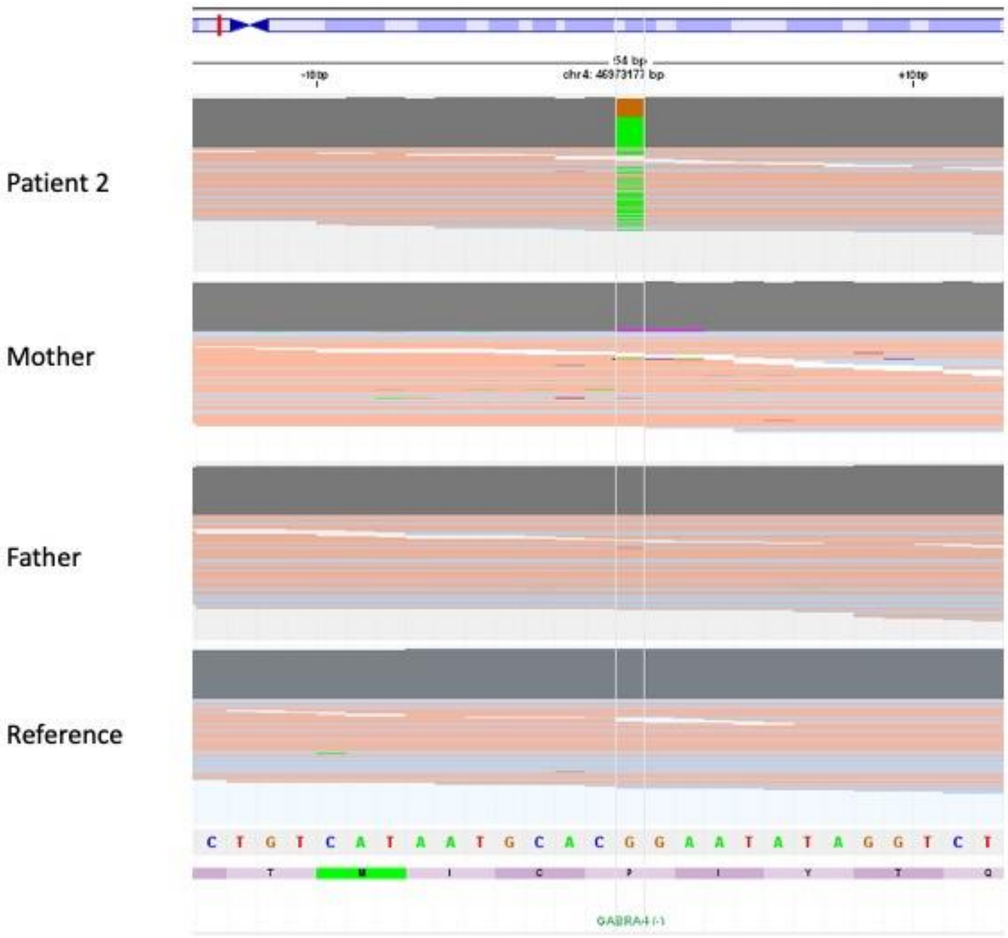

Supplementary figure 5. EEG of patient 3. (A) Background activity with diffuse slowing and frequent bilateral occipital spikes. (B) One of the patient's typical atonic seizures. There is a diffuse slow wave followed by an electro-decrement with overriding, posterior predominant, fast activity. (C) EEG findings during seizure consisting of behavioral arrest, with seizure onset. (D) Seizure occurring bifrontally with evolution through the bilateral central regions, followed by (E) offset.

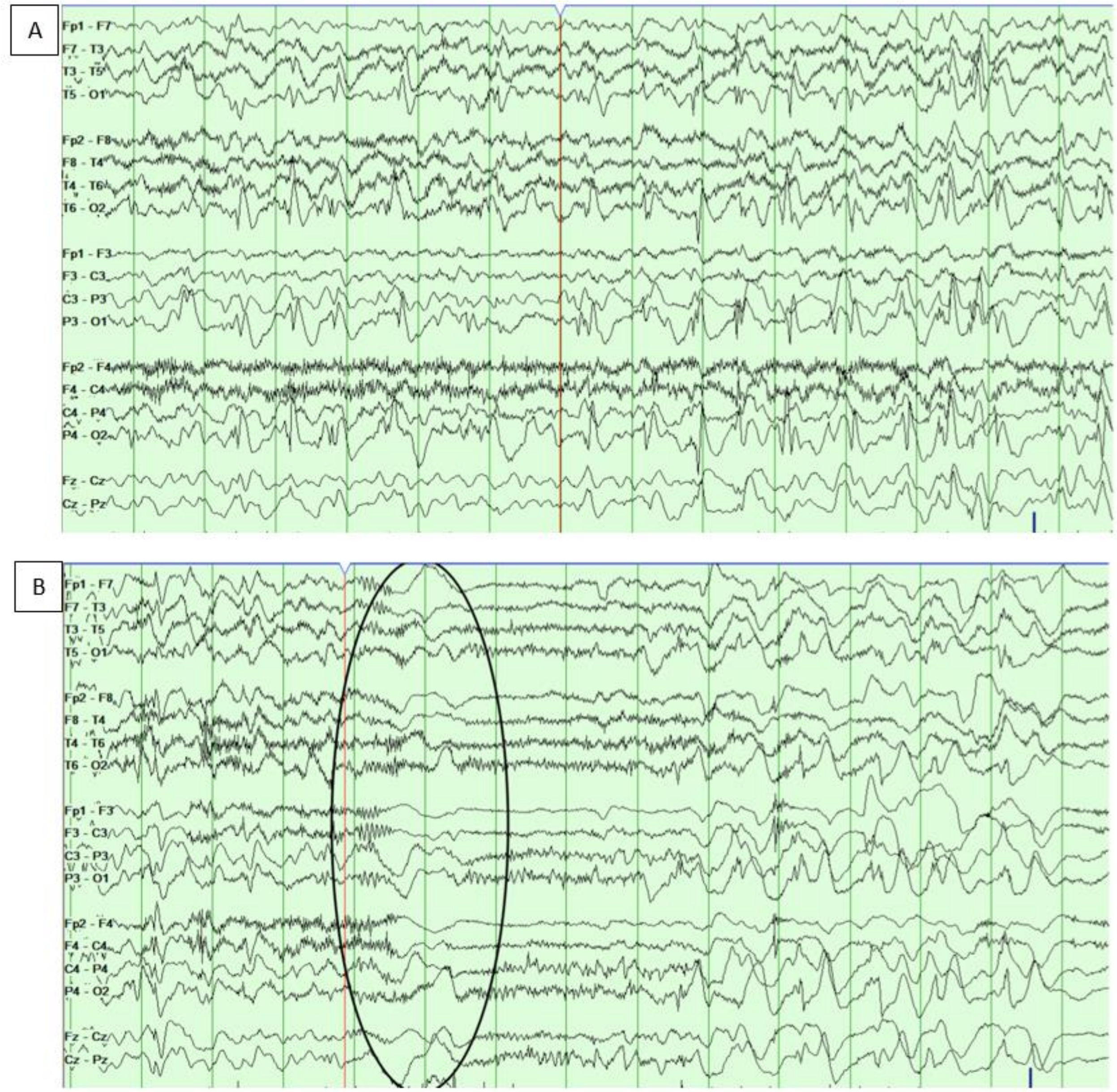

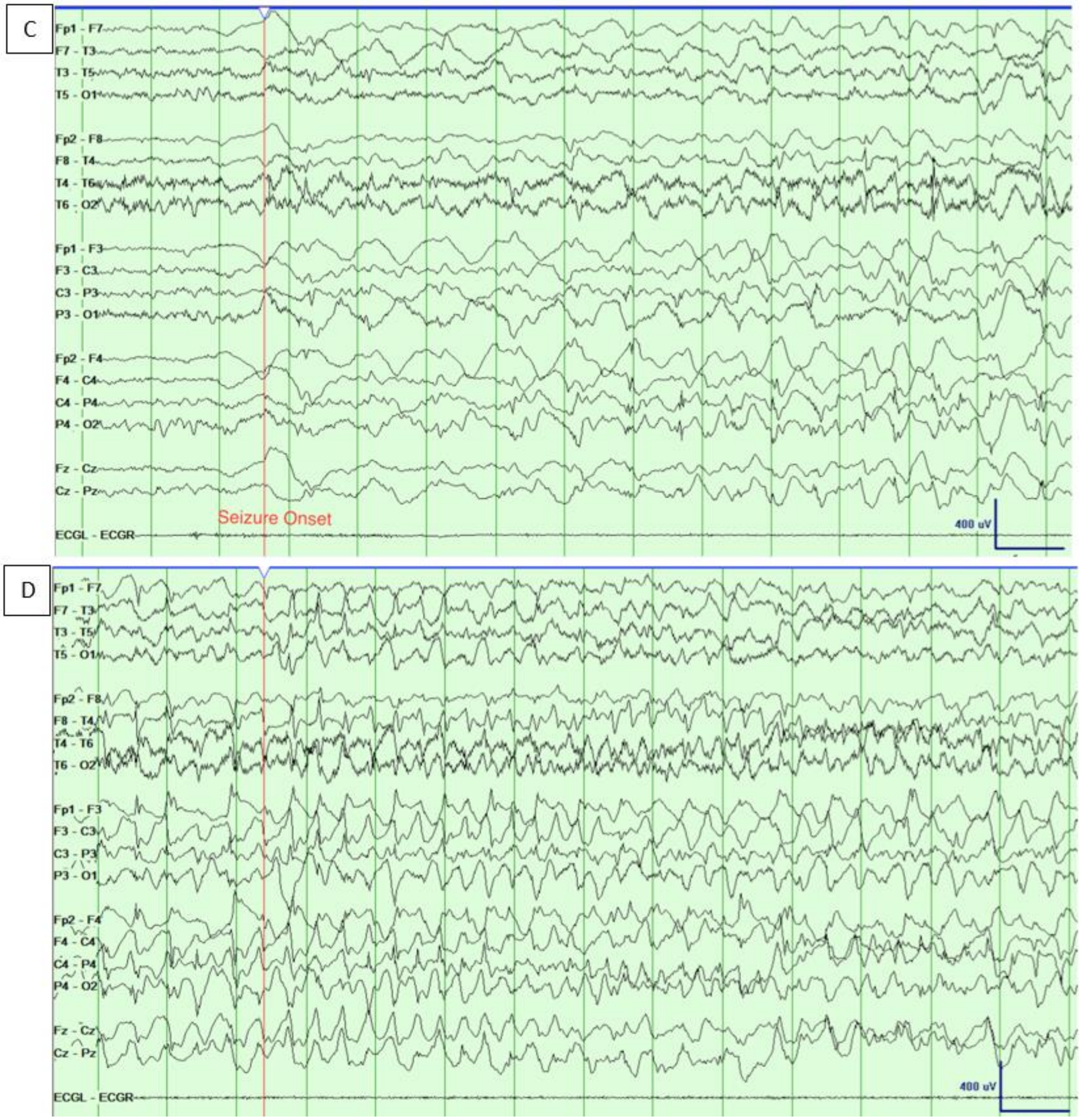
C
Fp1 - F7
F7 - T3
T3 - T5
T5 - O1
Fp2 - F8
F8 - T4
T4 - T6
T6 - O2
Fp1 - F3
F3 - C3
C3 - P3
P3 - O1
Fp2 - F4
F4 - C4
C4 - P4
P4 - O2
Fz - Cz
Cz - Pz
Seizure Onset
ECGL - ECGR
400 uV
D
Fp1 - F7
F7 - T3
T3 - T5
T5 - O1
Fp2 - F8
F8 - T4
T4 - T6
T6 - O2
Fp1 - F3
F3 - C3
C3 - P3
P3 - O1
Fp2 - F4
F4 - C4
C4 - P4
P4 - O2
Fz - Cz
Cz - Pz
ECGL - ECGR
400 uV

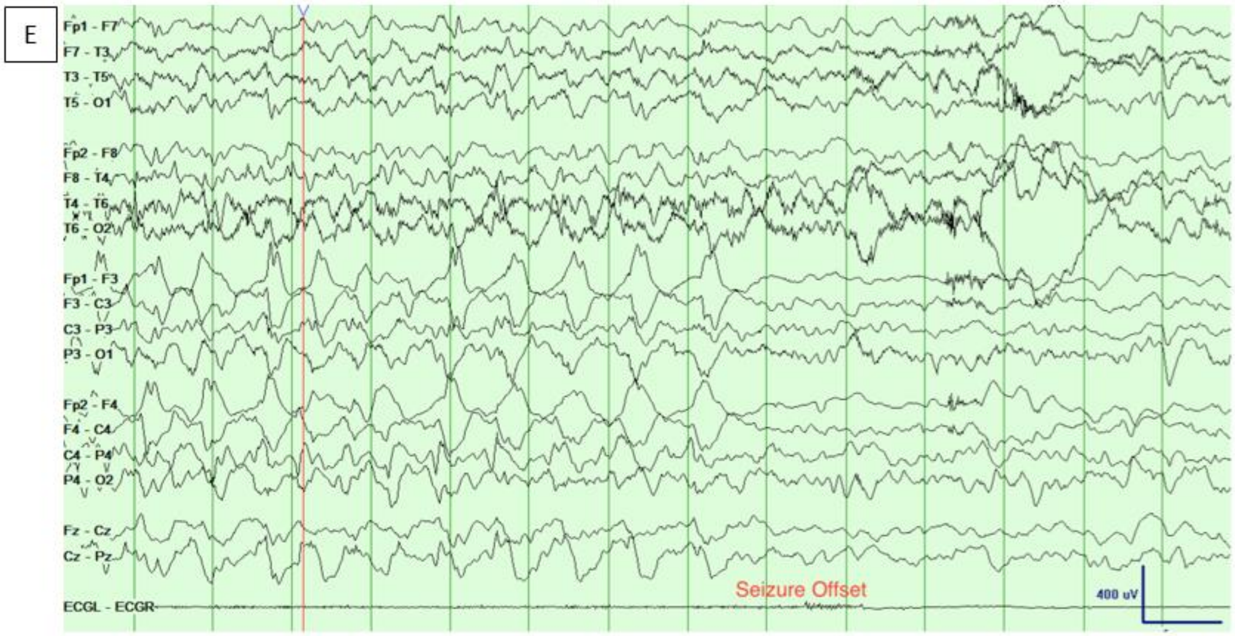

Supplementary figure 6. Brain MRI of patient 3 showing right frontal increased FLAIR signal, consistent with focal cortical dysplasia.

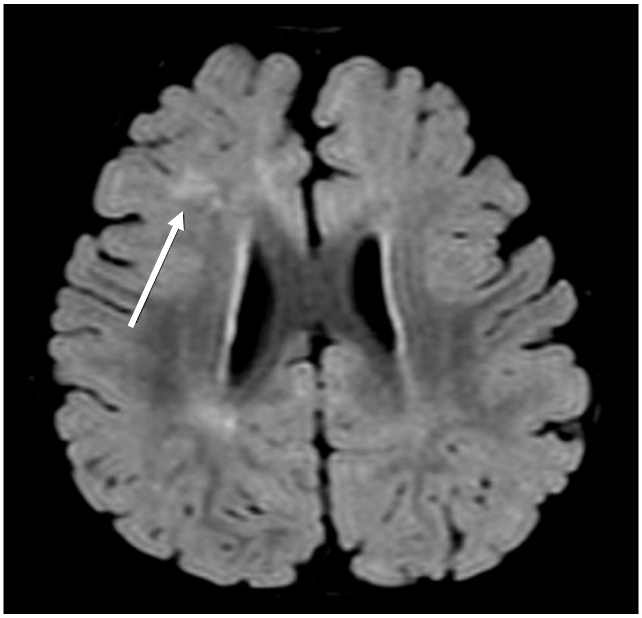

Supplementary figure 7. Integrated Genome Browser (IGV) views of the c.899C>A (p.T300N) variant in patient 3 and the parents. The variant was observed at an allele fraction of 16% in the patient (7 variant reads, and total coverage at this nucleotide being 44).

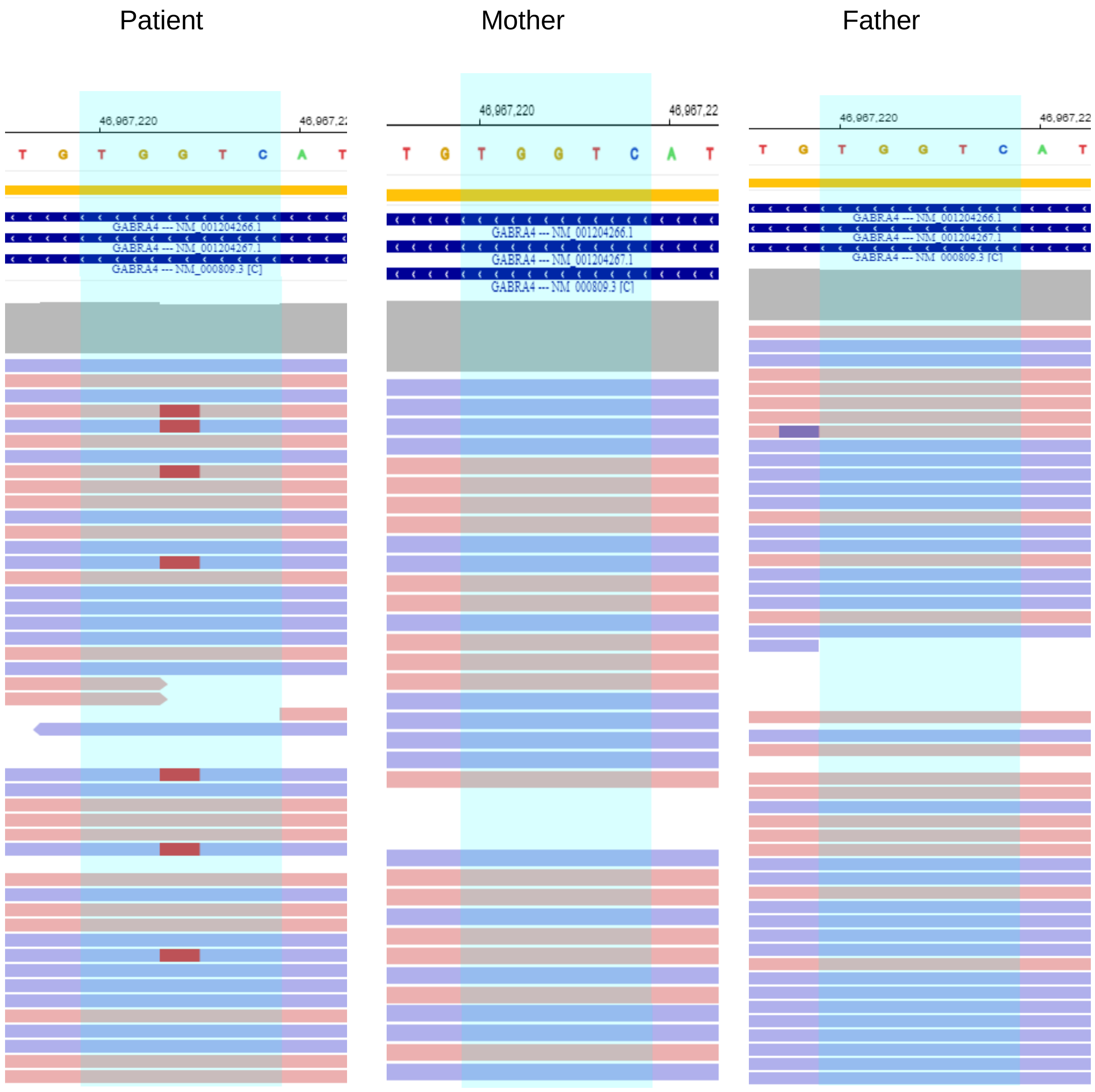

Supplementary figure 8. Bi-directional Sanger sequence showing the c.899C>A (p.T300N) variant in whole blood and oral mucosa of patient 3. An unrelated control sample negative for this variant was also sequenced. The nucleotide affected by the variant is highlighted in black and the green arrows indicate the presence of both wild-type and variant alleles, with the latter being smaller in height and therefore indicative of mosaicism. Quantification of peak heights showed that this variant was present at an allele fraction of approximately 9.2% in blood and 19.9% in oral mucosa. See supplementary table 1.

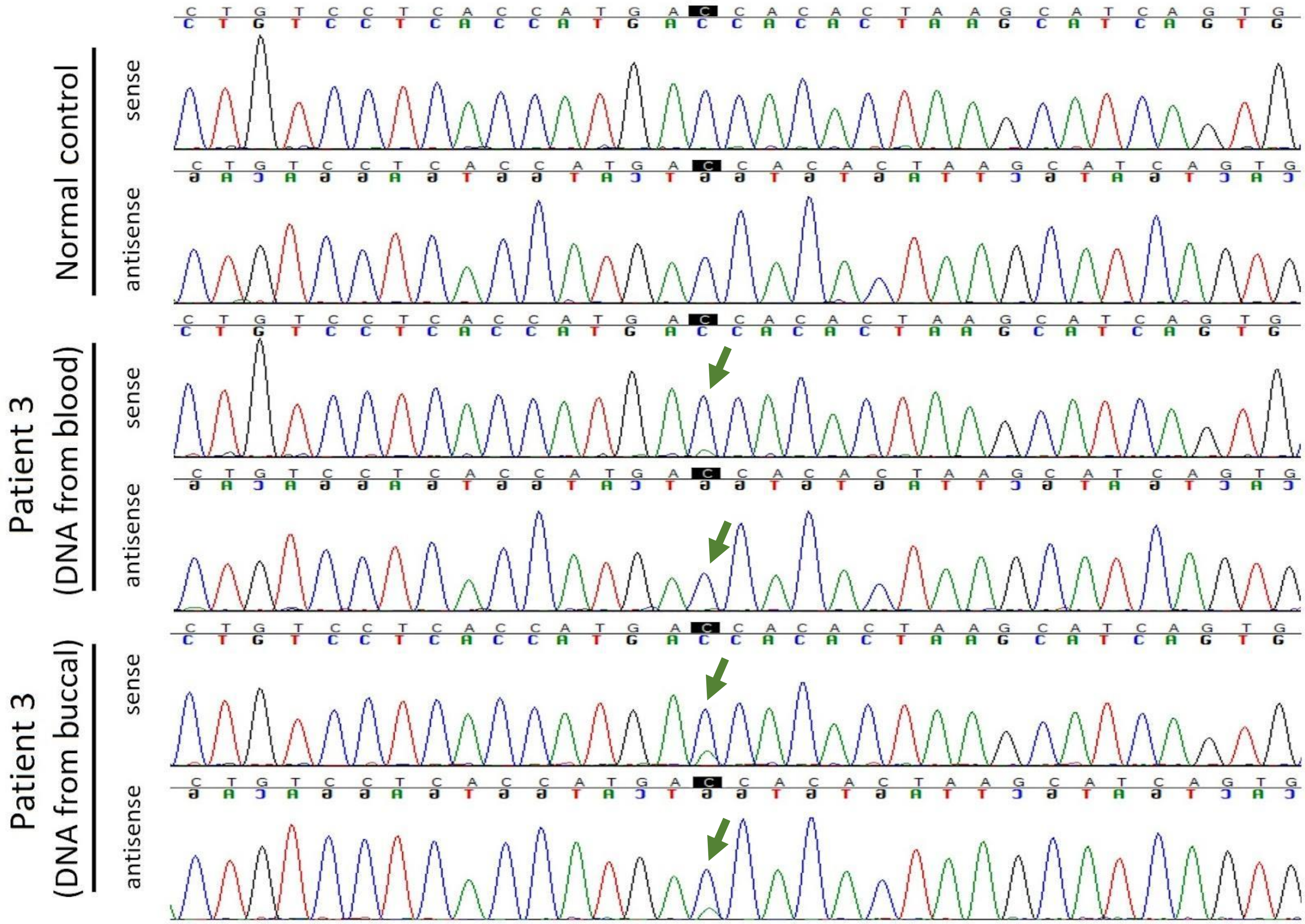

Supplementary figure 9. EEGs of patient 4 showing normal activity. The background frequency is normal, specifically in the low-normal range for age in A and B.

A

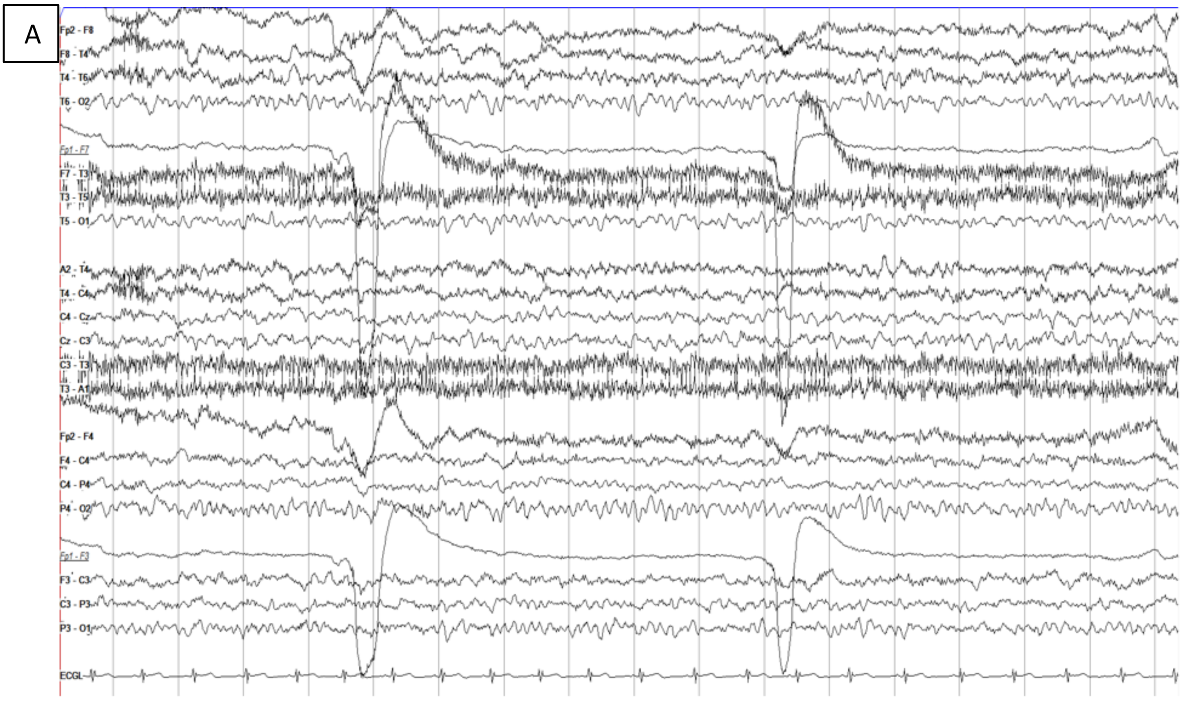

B

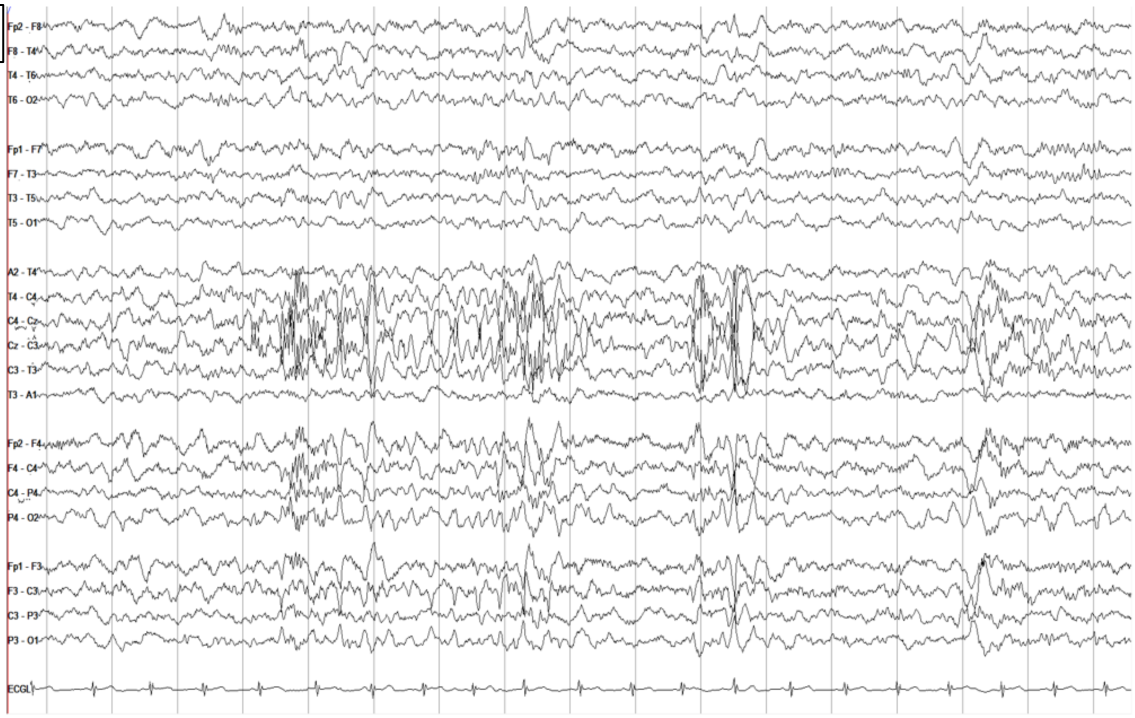

Supplementary figure 10. Integrated Genome Browser (IGV) views of the c.634G>A (p.V212I) variant in patient 4 and the mother.

Patient

Mother

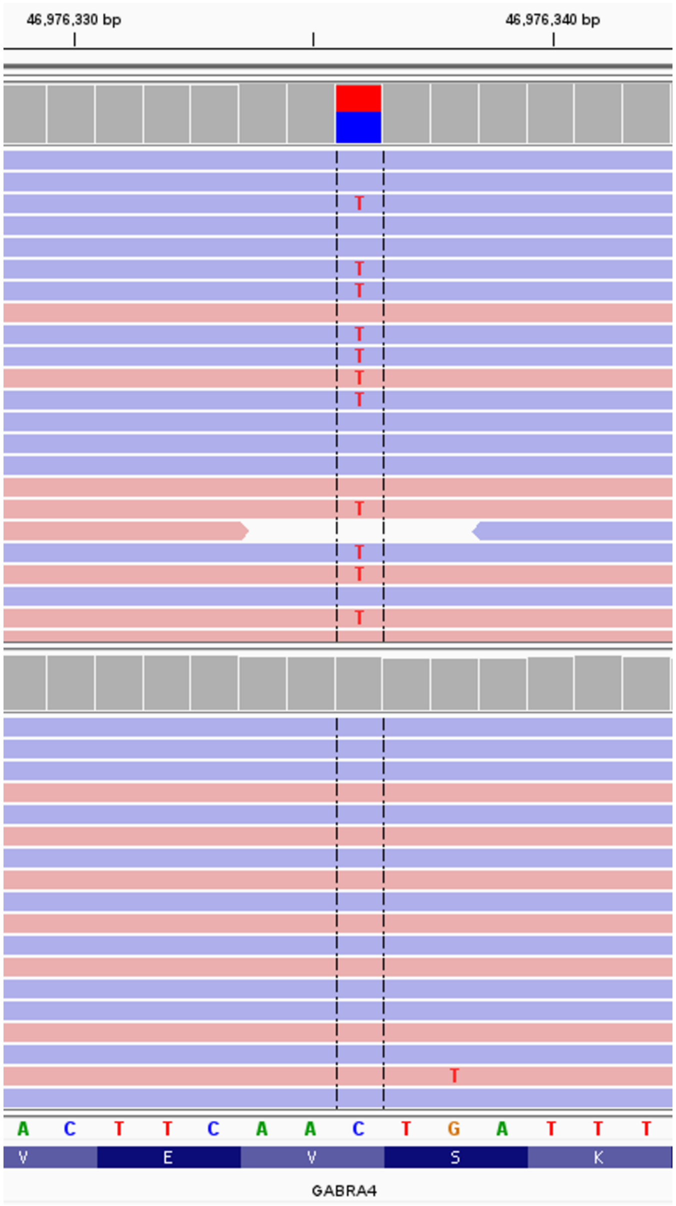

Supplementary figure 11. Bi-directional Sanger sequencing of the father of patient 4 with c.634G>A (p.V212I) showed that this variant was absent in the father.

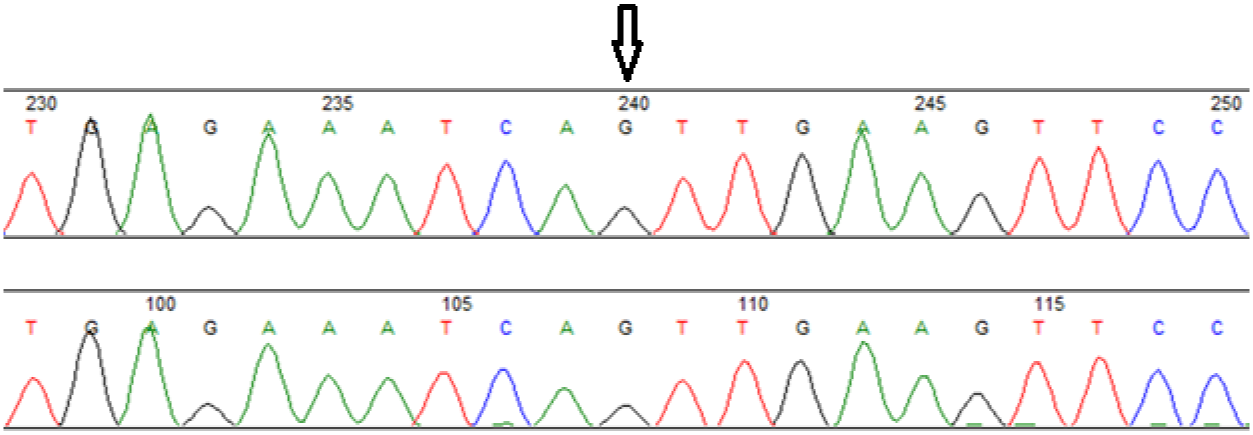

Supplementary table 1. Estimation of the level of mosaicism of the c.899C>A (p.T300N) variant in whole blood and oral mucosa of patient 3 by bi-directional Sanger sequencing. The height of the peaks for the normal allele and the variant allele were measured using the Mutation Quantifier Function in Mutation Surveyor v5.0.1.

**Sample name**	**Strand**	**Normal_ height**	**Variant_ height**	**Normal_ percent**	**Variant_ percent**	**Average _variant _percent**
Normal control	Sense	664	0	100.0%	0.0%	0.0%
Normal control	Antisense	544	0	100.0%	0.0%	
DNA from blood	Sense	688	83	89.2%	10.8%	9.2%
DNA from blood	Antisense	441	36	92.5%	7.6%	
DNA from buccal swab	Sense	703	182	79.4%	20.6%	19.9%
DNA from buccal swab	Antisense	537	127	80.9%	19.1%	

Supplementary methods for molecular dynamics studies

Model preparation

We used the pre-open cryo-EM structure of GABRα4β3δ (PDB ID:7QN9) that was solved at a resolution of 2.9 Å[1]. N-methyl and acetyl groups were introduced to neutralize the interfaces at the extracellular and intracellular loops at missing loops (The missing residues are: α4: M1-T45, E349-G514, and D545-M554; β3: M1-G32, G335-T443, and N472; δ: M1-N43, Y337-D423, and M452) using PyMOL version 2.3.0[2]. To ensure proper membrane insertion, the structure was aligned to the β3-subunits of the human GABRα1β3γ2 (PDB ID: 6i53)[3] as present in the OPM database[4]. The three histamine molecules bound to the cryo-EM structure were double protonated, therefor carrying a positive charge. The force field parameters for histamine were acquired using the General Amber Force Field (GAFF)[5] and ACPYPE[6]. The R.E.D server was used to derive partial charges[7]. The GABRα4β3δ receptor in complex with the three histamine molecules was embedded in an asymmetric complex phospholipid bilayer with the following composition in mol %: Upper leaflet: 60% (1-palmitoyl-2-oleoyl-phosphatidylcholine, POPC), 30% (cholesterol, CHOL), 10% (1-palmitoyl-2-oleoyl-phosphatidylethylamine, POPE); Lower leaflet: 20% (POPC), 35% (POPE), 15% (1-palmitoyl-2-oleoyl-phosphatidylserine, POPS), 30% (CHOL)[8] and surrounded by water and 150 mM NaCl. Point mutations in the α4-subunit were created using the Wizard Mutagenesis tool provided by PyMOL. By default, all glutamate and aspartate sidechains were charged. Histidine residues were treated as neutral and protonated at the epsilon nitrogen, which is the most abundant configuration at physiological pH.

Coarse-grained simulations

To create an equilibrated lipid environment including water and ions, we transformed three independently created GABRα4β3δ receptor systems into a coarse-grained (CG) representation using the MARTINI force field[9,10]. Each system was equilibrated by a 10 µs long simulation using Gromacs version 2019.3[11], hence allowing the membrane to extensively equilibrate, while applying position restraints (500 kJ/mol/nm) to the GABRα4β3δ receptor to prevent conformational changes. The simulation box had the dimensions 13.0 x 13.0 x 15.0 nm.

All atom simulations

To describe the protein, water, and ions in the all atom representation, the amber99sb-ildn force field was used for protein, water and ions[12], while the membrane was described using Slipid[13–16]. Gromacs 2022.3 was used to conduct all-atom simulations. The system equilibrated by the 10 µs CG simulations were transformed to an atomistic (all atom) representation using the backward.py script[17]. The GABRα4β3δ structure in complex with the three histamines was used to replace it with the original GABRα4β3δ structure to remove spurious distortion by the double transformation procedure. Afterwards, the structure underwent 200 rounds of energy minimization using the steepest descent algorithm, each comprising 10 minimisation steps. To further optimize the system due to remaining shortcomings introduced by the backmapping procedure, we performed two short simulations of 1000 steps at 1K with efficient energy coupling, while restraining the protein with 5000 kJ/mol/nm. Next, the system underwent

an equilibration procedure using a 4-step protocol that gradually reduces the position restraints on both the Cα atoms of GABRα4β3δ and histamines (1000, 100, 10, 1 kJ/mol/nm). MD production runs were carried out for 500 ns each. The temperature was kept at 310 K using the v-rescale thermostat (τ = 0.5 ps)[18] by independently coupling solvent, membrane, and protein in complex with histamines to the head bath. To keep the pressure steady at 1 bar, we utilized the Parrinello-Rahman barostat[19] in a semi isotropic fashion using a coupling constant of 20.1 ps. The smooth particle mesh Ewald method[20] was used to describe long-range electrostatic interactions with a cutoff of 0.9 nm. The Van der Waals interactions were described using the Lennard Jones potentials using a cutoff of 0.9 nm. Long-range correction for energy and pressure were applied. Coordinates of all atoms were recorded at every 25 ps.

All atom simulations analysis

The raw MD trajectories were processed and analyzed using Gromacs version 2022.3 and MD Analysis version 2.0.0[21,22]. Spatial occupancy of water molecules was calculated with the VolMap tool of VMD[23]. Plots were generated using the R package and R studio.

Supplementary references

1. Sente A, Desai R, Naydenova K, et al. Differential assembly diversifies GABAA receptor structures and signalling. *Nature*. 2022;604(7904):190-194. doi:10.1038/s41586-022-04517-3

2. DeLano WL. The PyMOL Molecular Graphics System, Version 1.2r3pre, Schrödinger, LLC. Published online 2002.

3. Laverty D, Desai R, Uchański T, et al. Cryo-EM structure of the human α1β3γ2 GABAA receptor in a lipid bilayer. *Nature*. 2019;565(7740):516-520. doi:10.1038/s41586-018-0833-4

4. Lomize MA, Pogozheva ID, Joo H, Mosberg HI, Lomize AL. OPM database and PPM web server: resources for positioning of proteins in membranes. *Nucleic Acids Res*. 2012;40(Database issue):D370-6. doi:10.1093/nar/gkr703

5. Wang J, Wolf RM, Caldwell JW, Kollman PA, Case DA. Development and testing of a general amber force field. *J Comput Chem*. 2004;25(9):1157-1174. doi:10.1002/jcc.20035

6. Sousa da Silva AW, Vranken WF. ACPYPE - AnteChamber PYthon Parser interfacE. *BMC Res Notes*. 2012;5:367. doi:10.1186/1756-0500-5-367

7. Vanquelef E, Simon S, Marquant G, et al. R.E.D. Server: a web service for deriving RESP and ESP charges and building force field libraries for new molecules and molecular fragments. *Nucleic Acids Res*. 2011;39(Web Server issue):W511-7. doi:10.1093/nar/gkr288

8. Hedger G, Koldsø H, Chavent M, Siebold C, Rohatgi R, Sansom MSP. Cholesterol Interaction Sites on the Transmembrane Domain of the Hedgehog Signal Transducer and Class F G Protein-Coupled Receptor Smoothened. *Structure*. 2019;27(3):549-559.e2. doi:10.1016/j.str.2018.11.003

9. Monticelli L, Kandasamy SK, Periole X, Larson RG, Tieleman DP, Marrink SJ. The MARTINI Coarse-Grained Force Field: Extension to Proteins. *J Chem Theory Comput*. 2008;4(5):819-834. doi:10.1021/ct700324x

10. de Jong DH, Singh G, Bennett WFD, et al. Improved Parameters for the Martini Coarse-Grained Protein Force Field. *J Chem Theory Comput*. 2013;9(1):687-697. doi:10.1021/ct300646g

11. Abraham MJ, Murtola T, Schulz R, et al. GROMACS: High performance molecular simulations through multi-level parallelism from laptops to supercomputers. *SoftwareX*. 2015;1-2:19-25. doi:10.1016/j.softx.2015.06.001

12. Lindorff-Larsen K, Piana S, Palmo K, et al. Improved side-chain torsion potentials for the Amber ff99SB protein force field. *Proteins*. 2010;78(8):1950-1958. doi:10.1002/prot.22711

13. Jämbeck JPM, Lyubartsev AP. An Extension and Further Validation of an All-Atomistic Force Field for Biological Membranes. *J Chem Theory Comput*. 2012;8(8):2938-2948. doi:10.1021/ct300342n

14. Jämbeck JPM, Lyubartsev AP. Another Piece of the Membrane Puzzle: Extending Slipids Further. *J Chem Theory Comput*. 2013;9(1):774-784. doi:10.1021/ct300777p

15. Ermilova I, Lyubartsev AP. Extension of the Slipids Force Field to Polyunsaturated Lipids. *J Phys Chem B*. 2016;120(50):12826-12842. doi:10.1021/acs.jpcb.6b05422

16. Grote F, Lyubartsev AP. Optimization of Slipids Force Field Parameters Describing Headgroups of Phospholipids. *J Phys Chem B*. 2020;124(40):8784-8793. doi:10.1021/acs.jpcb.0c06386

17. Wassenaar TA, Pluhackova K, Böckmann RA, Marrink SJ, Tieleman DP. Going Backward: A Flexible Geometric Approach to Reverse Transformation from Coarse Grained to Atomistic Models. *J Chem Theory Comput*. 2014;10(2):676-690. doi:10.1021/ct400617g

18. Bussi G, Donadio D, Parrinello M. Canonical sampling through velocity rescaling. *J Chem Phys*. 2007;126(1):014101. doi:10.1063/1.2408420

19. Parrinello M, Rahman A. Polymorphic transitions in single crystals: A new molecular dynamics method. *J Appl Phys*. 1981;52(12):7182-7190. doi:10.1063/1.328693

20. Darden T, York D, Pedersen L. Particle mesh Ewald: An N ⋅log(N) method for Ewald sums in large systems. *J Chem Phys*. 1993;98(12):10089-10092. doi:10.1063/1.464397

21. Michaud-Agrawal N, Denning EJ, Woolf TB, Beckstein O. MDAnalysis: a toolkit for the analysis of molecular dynamics simulations. *J Comput Chem*. 2011;32(10):2319-2327. doi:10.1002/jcc.21787

22. Gowers R, Linke M, Barnoud J, et al. MDAnalysis: A Python Package for the Rapid Analysis of Molecular Dynamics Simulations. In: *Proceedings of the 15th Python in Science Conference*. ; 2016:98-105. doi:10.25080/Majora-629e541a-00e

23. Humphrey W, Dalke A, Schulten K. VMD: visual molecular dynamics. *J Mol Graph*. 1996;14(1):33-38, 27-28. doi:10.1016/0263-7855(96)00018-5
